# Screening Key Genes and Biological Pathways in Nasopharyngeal Carcinoma by Integrated Bioinformatics Analysis

**DOI:** 10.3390/ijms232415701

**Published:** 2022-12-11

**Authors:** Junhu Tai, Jaehyung Park, Munsoo Han, Tae Hoon Kim

**Affiliations:** 1 Department of Otorhinolaryngology-Head & Neck Surgery, College of Medicine, Korea University, Seoul 02841, Republic of Korea; junhu69@korea.ac.kr (J.T.); godz131@korea.ac.kr (J.P.); mshan35@gmail.com (M.H.); 2 Mucosal Immunology Institute, College of Medicine, Korea University, Seoul 02841, Republic of Korea

**Keywords:** nasopharyngeal carcinoma, bioinformatics, genes

## Abstract

The purpose of this study was to identify the hub genes and biological pathways of nasopharyngeal carcinoma (NPC) through bioinformatics analysis and potential new therapeutic targets. In this study, three datasets were downloaded from the Gene Expression Omnibus (GEO), and differentially expressed genes (DEGs) between NPC and normal tissues were analyzed using the GEO2R online tool. Volcano and heat maps of the DEGs were visualized using the hiplot database. Gene ontology (GO) and the Kyoto Encyclopedia of Genes and Genomes (KEGG) pathway enrichment analyses of the upregulated and downregulated DEGs were performed using the DAVID database. Finally, we established a protein-protein interaction (PPI) network using the STRING database and showed the differential expression of hub genes between the normal and tumor tissues. In all, 109,371,221 upregulated DEGs and 139,226,520 downregulated DEGs were obtained in datasets GSE40290, GSE61218, and GSE53819, respectively, and 18 common differential genes, named co-DEGs, were screened in the three datasets. The most abundant biological GO terms of the co-DEGs were inflammatory response et al. The KEGG pathway enrichment analysis showed that co-DEGs mainly participated in the interleukin (IL)-17 signaling pathway et al. Finally, we identified four hub genes using PPI analysis and observed that three of them were highly expressed in tumor tissues. In this study, the hub genes of NPC, such as PTGS2, and pathways such as IL-17 signaling, were identified through bioinformatics analysis, which may be potential new therapeutic targets for NPC.

## 1. Introduction

Nasopharyngeal carcinoma (NPC) is an aggressive head and neck cancer that forms in the tissues of the nasopharynx with high malignancy, which often occurs in the pharyngeal recess, and is relatively rare compared with other cancers [1]. In 2018, approximately 130,000 new NPC cases and 73,000 related deaths were reported in Southeast Asia [2]. The occurrence and development of NPC are related to various factors, including Epstein-Barr virus (EBV) infection [3]. EBV-associated NPC is extremely sensitive to radiation therapy, while squamous histological subtypes are much less sensitive. There are also significant differences in clinical manifestations and treatment responses between undifferentiated and squamous variants [4]. Concurrent chemoradiotherapy is one of the main treatment methods for NPC; however, the emergence of chemotherapy resistance and high incidence of adverse events limit its application [5].

Bioinformatics is an analytical method that uses mathematical, statistical, and computational methods to process and analyze biological data, which differs from traditional laboratory work [6]. For example, Song et al. analyzed the key genes of NPC using bioinformatics [7], and Yue et al. expounded on the differentially expressed genes (DEGs) in NPC tissues and their correlation with the recurrence and metastasis of NPC [8]. Although many studies have focused on identifying biomarkers related to NPC, a more comprehensive analysis is needed to explore better molecular targets to treat NPC and clarify its biological pathways.

The purpose of this study was to screen DEGs and co-DEGs in multiple datasets by analyzing NPC-related datasets in the Gene Expression Omnibus (GEO) database [9], and to conduct gene ontology (GO) terminology and Kyoto Encyclopedia of Genes and Genomes (KEGG) pathway analysis. Finally, hub genes were obtained by constructing a protein-protein interaction (PPI) network, and the expression of hub genes in NPC was studied.

## 2. Results

### 2.1. Identification of DEGs in the Three GEO Data Sets

**Figure 1:**
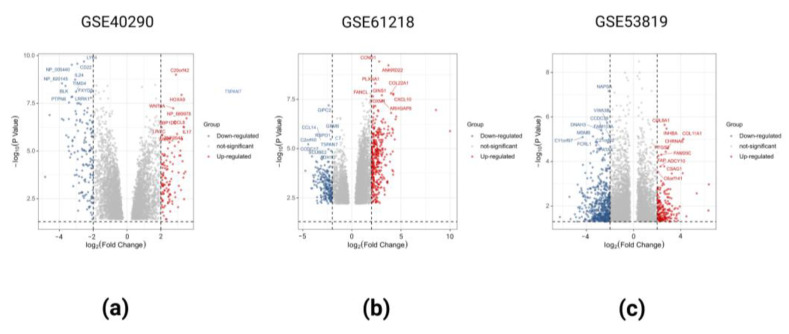
Volcano maps of DEGs in GSE40290 (**a**), GSE61218 (**b**), and GSE53819 (**c**).

**Figure 2:**
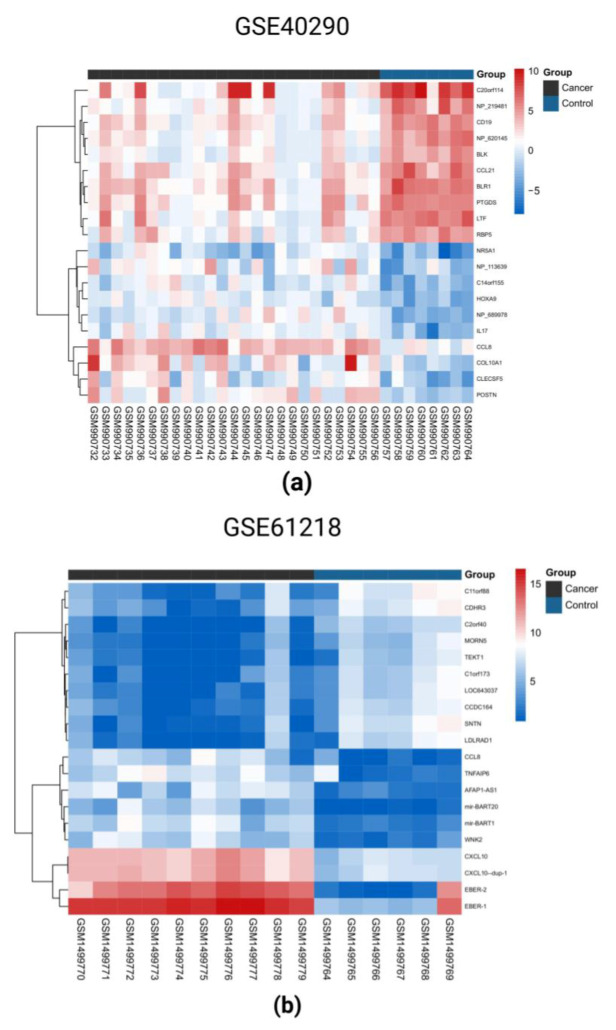

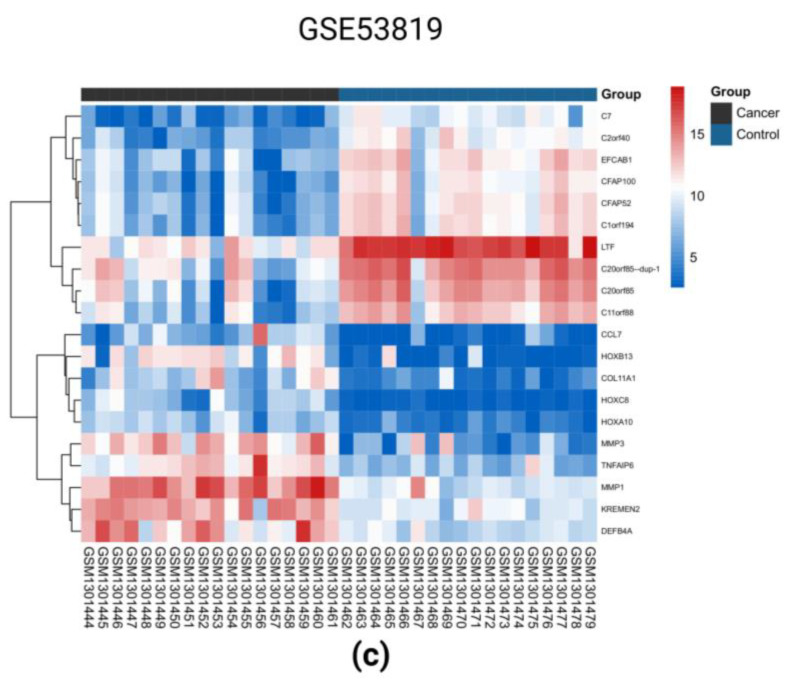
Heat maps of the top 20 DEGs in GSE40290 (**a**), GSE61218 (**b**), and GSE53819 (**c**).

**Figure 3:**
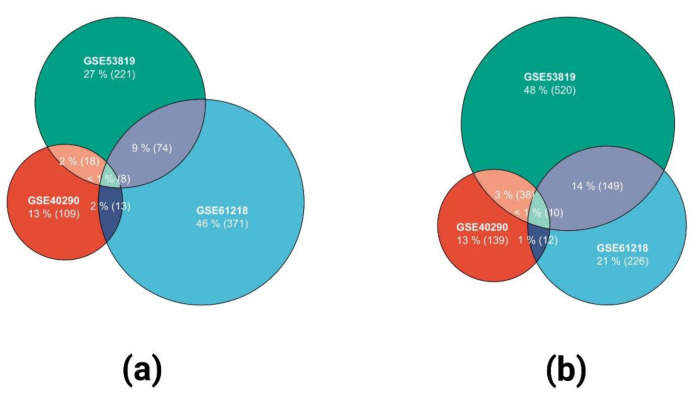
Wayne diagram of the upregulated DEGs (**a**) and downregulated DEGs (**b**).

DEGs were defined as follows: when *p* < 0.05 and fold change > 2, DEGs were upregulated; when *p* < 0.05 and fold change < 2, DEGs were downregulated. After online analysis using GEO2R, 109,371,221 upregulated DEGs and 139,226,520 downregulated DEGs were obtained in datasets GSE40290, GSE61218, and GSE53819, respectively. The corresponding volcano maps of GSE40290 (Figure 1a), GSE61218 (Figure 1b), and GSE53819 (Figure 1c) are shown. Heat maps of the top 20 DEGs in GSE40290 (Figure 2a), GSE61218 (Figure 2b), and GSE53819 (Figure 2c) were generated. By cross-analyzing the three datasets, eight co-upregulated genes (Figure 3a) and 10 co-downregulated genes (Figure 3b) were identified, and a scale Wayne diagram was used to visualize the data.

### 2.2. GO and KEGG Pathway Enrichment Analysis of Upregulated DEGs

**Figure 4:**
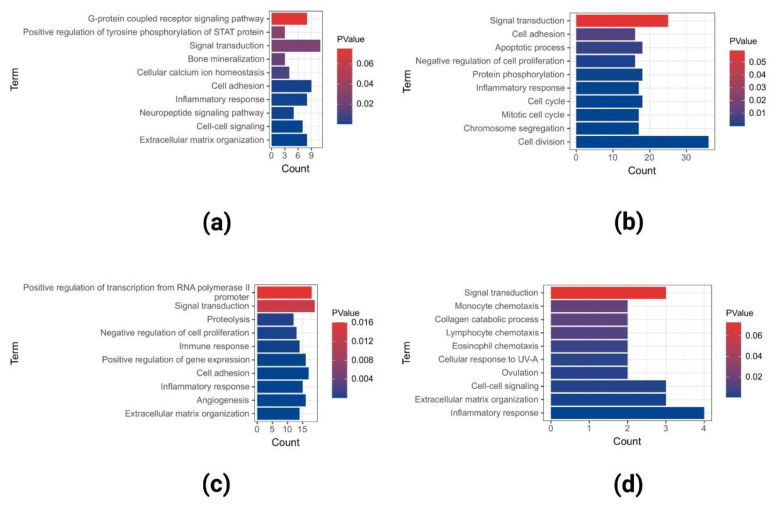
GO analyses of upregulated DEGs in GSE40290 (**a**), GSE61218 (**b**), GSE53819 (**c**), and co-upregulated DEGs (**d**) in the three datasets.

We used GO and KEGG pathway enrichment analyses to characterize the functional effects of each dataset and co-DEGs in the three datasets. The most abundant GO terms in the upregulated DEGs of GSE40290 included signal transduction, cell adhesion, extracellular matrix organization, and so on (Figure 4a). The most abundant GO terms in the upregulated DEGs of GSE61218 included cell division, signal transduction, cell cycle, and so on (Figure 4b). The most abundant GO terms in the upregulated DEGs of GSE53819 included signal transduction, positive regulation of transcription from RNA polymerase II promoter, cell adhesion, and so on (Figure 4c). The most abundant GO terms in the co-upregulated DEGs in the three datasets included inflammatory response, extracellular matrix organization, cell-cell signaling, and so on (Figure 4d).

**Figure 5:**
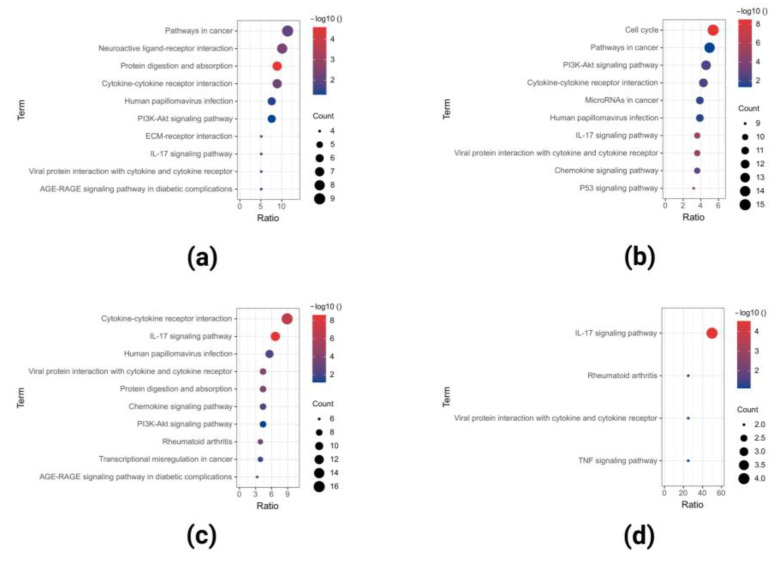
KEGG analyses of the upregulated DEGs in GSE40290 (**a**), GSE61218 (**b**), GSE53819 (**c**), and co-upregulated DEGs (**d**) in the three datasets.

KEGG analysis results showed that the upregulated DEGs of GSE40290 were significantly enriched in pathways in cancer, neuroactive ligand-receptor interaction, protein digestion and absorption, and so on (Figure 5a); upregulated DEGs of GSE61218 were significantly enriched in the cell cycle, pathways in cancer, PI3K-Akt signaling pathway, and so on (Figure 5b); upregulated DEGs of GSE53819 were significantly enriched in cytokine-cytokine receptor interaction, IL-17 signaling pathway, human papillomavirus infection, and so on (Figure 5c); and co-upregulated DEGs in the three datasets were significantly enriched in the IL-17 signaling pathway, rheumatoid arthritis, viral protein interaction with cytokine and cytokine receptor, and so on (Figure 5d).

### 2.3. GO and KEGG Pathway Enrichment Analyses of Downregulated DEGs

**Figure 6:**
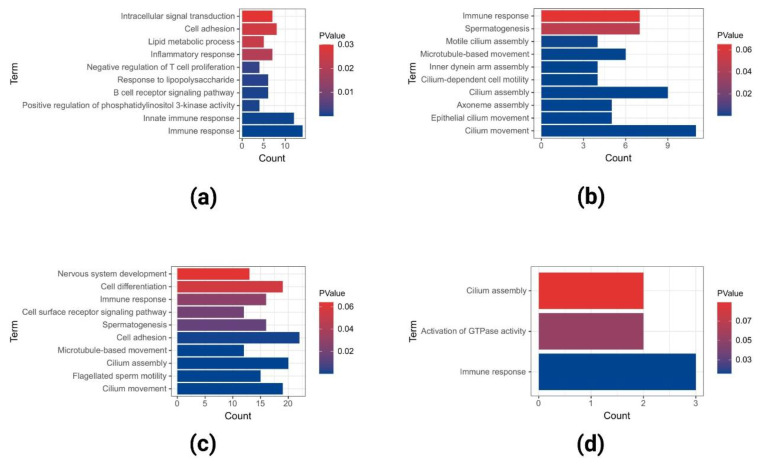
GO analyses of downregulated DEGs in GSE40290 (**a**), GSE61218 (**b**), GSE53819 (**c**), and co-downregulated DEGs (**d**) in the three datasets.

The most abundant GO terms in the downregulated DEGs of GSE40290 included immune response, innate immune response, cell adhesion, and so on (Figure 6a). The most abundant GO terms in the downregulated DEGs of GSE61218 included cilium movement, cilium assembly, spermatogenesis, and so on (Figure 6b). The most abundant GO terms in the downregulated DEGs of GSE53819 included cell adhesion, cilium assembly, cilium movement, and so on (Figure 6c). The most abundant GO terms in the co-downregulated DEGs in the three datasets included immune response, activation of GTPase activity, and cilium assembly (Figure 6d).

**Figure 7:**
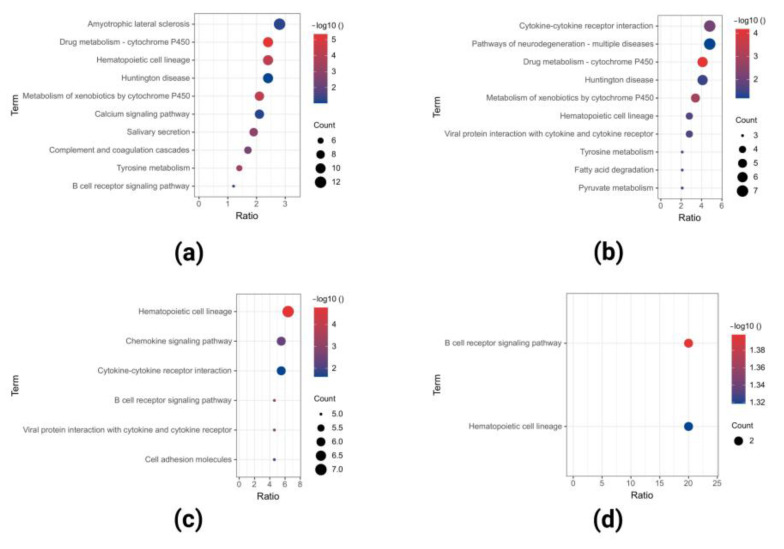
KEGG analyses of downregulated DEGs in GSE40290 (**a**), GSE61218 (**b**), GSE53819 (**c**), and co-downregulated DEGs (**d**) in the three datasets.

KEGG analysis results showed that the downregulated DEGs of GSE40290 were significantly enriched in amyotrophic lateral sclerosis, drug metabolism-cytochrome P450, hematopoietic cell lineage, and so on (Figure 7a); downregulated DEGs of GSE61218 were significantly enriched in cytokine-cytokine receptor interaction, pathways of neurodegeneration-multiple diseases, drug metabolism-cytochrome P450, and so on (Figure 7b); downregulated DEGs of GSE53819 were significantly enriched in hematopoietic cell lineage, chemokine signaling pathway, cytokine-cytokine receptor interaction, and so on (Figure 7c); and co-downregulated DEGs in the three datasets were most significantly enriched in the B-cell receptor signaling pathway and hematopoietic cell lineage (Figure 7d).

### 2.4. PPI Network Construction of Co-DEGs

**Figure 8:**
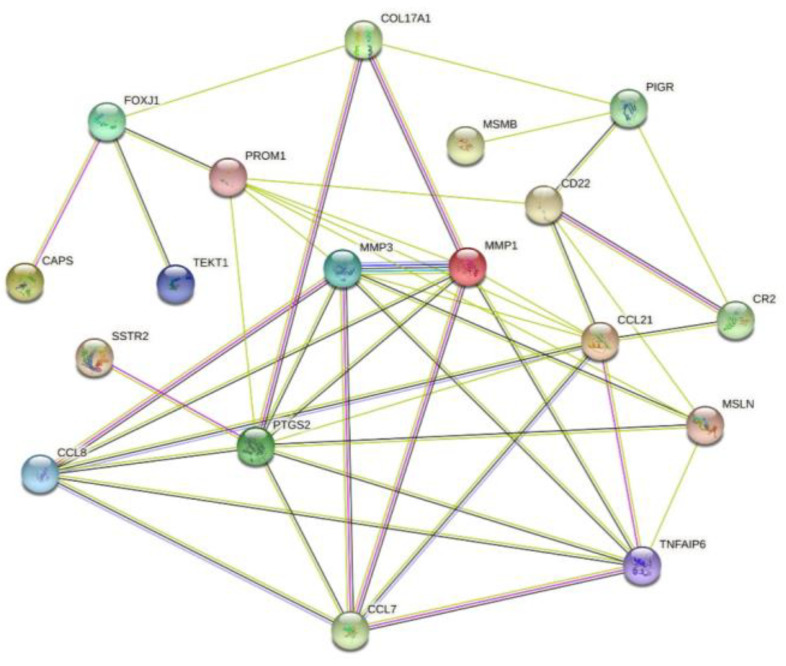
PPI network for co-DEGs in the three datasets.

We submitted eight co-upregulated DEGs and 10 co-downregulated DEGs in GSE40290, GSE61218, and GSE53819 to the STRING database for PPI analysis to identify hub genes. DEGs with connectivity greater than eight were selected as the hub genes, and four hub genes were identified, of which prostaglandin-endoperoxide synthase 2 (PTGS2) had a connectivity of 10; the degree of chemokine (C-C motif) ligand 21 (CCL21) was 9, degree of matrix metalloproteinase (MMP) 1 was 8, and degree of MMP3 was 8 (Figure 8).

### 2.5. Expression of Selected Hub Genes in Tumor Tissues

**Figure 9:**
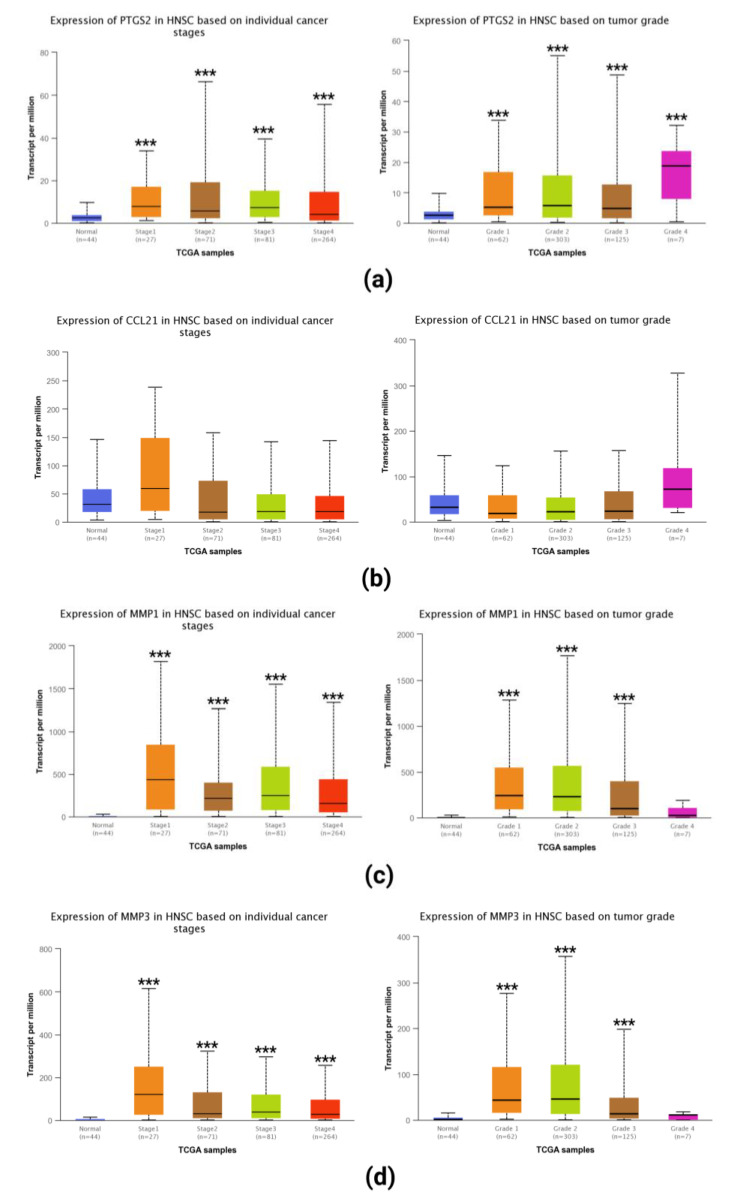
Different expressions of PTGS2 (**a**), CCL21 (**b**), MMP1 (**c**), and MMP3 (**d**) between normal and tumor tissues, ***: *p* < 0.001.

**Figure 10:**
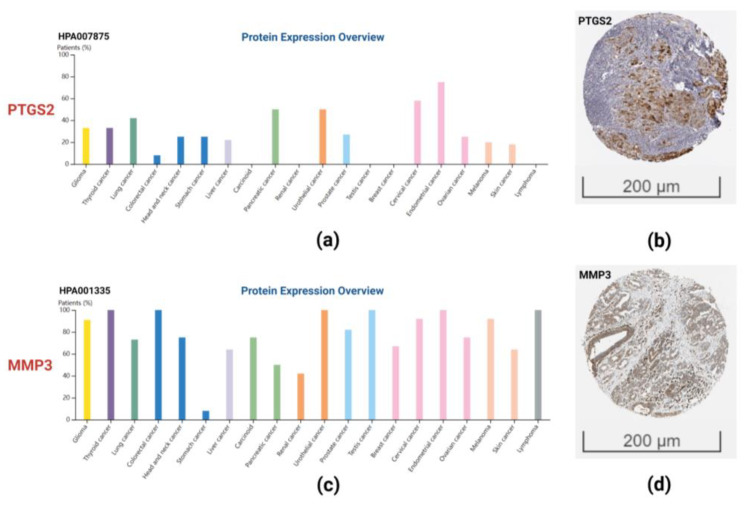
Expressions of PTGS2 in various cancer tissues (**a**), PTGS2 shows high staining in HSNC (**b**), expressions of MMP3 in various cancer tissues (**c**), and MMP3 also shows strong staining in HSNC (**d**).

The UALCAN database was used for the analysis of the TCGA head and neck squamous cancer (HNSC) dataset. We found that the PTGS2 showed higher expression in tumor tissues (Figure 9a). CCL21 did not show differential expression in normal and tumor tissues, and the expression of MMP1 (Figure 9c) and MMP3 (Figure 9d) showed higher expression in tumor tissues. Finally, we confirmed the expression of PTGS2 (Figure 10a) and MMP3 (Figure 10c) in various cancer tissues and high expression of PTGS2 (Figure 10b) and MMP3 (Figure 10d) in HNSC through Human Protein Atlas (HPA) database.

## 3. Discussion

Various cancers, including NPC, are among the major causes of human death worldwide. Globalization and an increase in various risk factors may further aggravate this situation. Bioinformatics methods are rapidly being developed for the analysis of biological data, especially in the analysis of large datasets, and have become an area of interest for researchers. Transforming biological data into knowledge through bioinformatics methods for study and analysis is more time-saving, efficient, and cost-effective than traditional methods [10]. In this study, we analyzed three datasets (GSE40290, GSE61218, and GSE53819) related to NPC using microarray data and identified 18 co-DEGs.

GO enrichment analysis showed that the most abundant GO terms in the co-upregulated DEGs in the three datasets included inflammatory response, extracellular matrix organization, and cell-cell signaling. Li et al. showed that the EBV M81 strain isolated from NPC-induced chronic inflammation in its target cells resulted in an increase in virus production. They explained the relationship between M81 virus replication and chemokines involved in inflammation and carcinogenesis [11]. Consistent with our results, inflammatory response was significantly enriched in the co-upregulated DEGs in their study. A clinical study that included 30 patients with NPC and 20 controls found that the process of tumor invasion and metastasis can be effectively reduced by controlling the activity of MMPs and extracellular matrix components [12]. This indicates that extracellular matrix components may play a promoting role in the development of NPC, which is consistent with our results. Exosomal microscopic RNAs from cancer cells play a key role in mediating cell-cell signaling and tumor microenvironment crosstalk. Lu et al. identified the inhibitory role of tumor-derived, exosome-related miR-9 in NPC tumorigenesis. In our results, the upregulated GO terms included cell-cell signaling, which is also similar to the result obtained by Lu et al. [13]. KEGG analysis results showed that the co-upregulated DEGs in the three datasets were significantly enriched in the IL-17 signaling pathway, rheumatoid arthritis and so on. The results of Wang et al. strongly revealed that IL-17 could activate the p38-NF-κB signaling pathway and promote the migration and invasion of NPC cells [14], which is consistent with our finding that co-upregulated DEGs were most enriched in the IL-17 signaling pathway. It has been pointed out that in rheumatoid arthritis, the response of antibodies to EBV induced cell antigens is significantly higher than that of healthy individuals [15], which is also consistent with our finding.

The most abundant GO terms in the co-downregulated DEGs in the three datasets were immune response, activation of GTPase activity, and cilium assembly. NPC tumorigenesis is significantly associated with genetic susceptibility. Recent epidemiological and large-scale genome-wide association studies have demonstrated an association between HLA class I genes and the risk of NPC. HLA class I gene coding is used to initiate the host immune response against malignant cells, but studies have found that high-risk people with several specific HLA haplotypes have low efficiency in the immune response to persistent EBV infection [16], which is consistent with our results. Jiang et al. showed that low GTPase expression is related to an increase in signal transduction, cell movement, and metastatic behavior of NPC cells [17]. This is consistent with the finding of our study that GO terms enriched in the co-downregulated DEGs included activation of GTPase activity. A previous study analyzing gene expression data from NPC and non-NPC nasopharyngeal tissues through a comprehensive pathway showed that the loss of function of the axonemal dynein complex in patients with NPC leads to impaired ciliary function, which in turn leads to poor mucociliary clearance and respiratory tract infection [18], which is also consistent with our results. The co-downregulated DEGs in the three datasets were most significantly enriched in the B-cell receptor signaling pathway and hematopoietic cell lineage. Morrison et al. showed that LMP2A expressed in most EBV-related tumors, including NPC, maintains virus latency by blocking the activation and signaling of B-cell receptors [19], which is consistent with our finding that co-downregulated DEGs were most enriched in the B-cell receptor signaling pathway. An article studying new aberrant methylation, differentially expressed genes and pathways in NPC pointed out that the hypermethylation/low-expression genes significantly enriched in hematopoietic cell lineage [20], which is also consistent with our finding.

Based on the PPI network, we screened four genes with the highest node degrees, including PTGS2, CCL21, MMP1, and MMP3. HNSC develops from the mucous lining of upper respiratory and digestive tract, including nasal cavity, paranasal sinus, oropharynx, larynx, and so on. However, NPC is a specific entity different from HNSC, the disease behavior of NPC is different from HNSC, and the treatment strategy is also different [21]. Since there is no separate NPC dataset in the TCGA database, we can only use the HNSC dataset for analysis. Our analysis using the HNSC dataset in the TCGA database revealed that the expression of PTGS2, MMP1, and MMP3 in tumor tissues was significantly higher. Through HPA database, we also found that PTGS2 and MMP3 are expressed in various cancers, including head and neck cancers. The results of antibody staining showed that PTGS2 and MMP3 were strongly expressed in HSNC. A recent meta-analysis identified the upregulation of PTGS2, MMP1, and MMP3 in NPC tissues, shows that the maladjustment of nasal epithelial barrier and maladjusted immune response are the key components in the pathogenesis of NPC [22]. Another previous meta-analysis found that the overexpression of PTGS2 was significantly associated with a low survival rate in patients with NPC [23]. A study detected high expression of PTGS2 in patients with NPC and distant metastasis and showed that PTGS2 was related to the migration and invasion of NPC cells, in addition to the low survival rate of patients with NPC [24]. A study involving 56 normal people and 114 patients with NPC was conducted to explore the correlation between PTGS2 gene polymorphism and the occurrence of NPC [25]. It was found that PTGS2 gene polymorphism was related to the susceptibility of NPC, and both smoking and EBV infection, which are the main risk factors of NPC, can affect PTGS2 gene polymorphism. Some studies have confirmed that the upregulation of MMP1 is related to lymph node metastasis in NPC [26]. Additionally, studies have shown that MMP1 is significantly associated with the risk of NPC [27]. Song et al. confirmed the upregulation of MMP1 in NPC tissues and cell lines by RT-qPCR and western blotting and found that knockdown of the MMP1 gene significantly inhibited cell proliferation and enhanced apoptosis [28]. A study that detected the mRNA and protein levels of MMP3 in NPC tissues and cells found that the concentration and enzymatic activity of MMP3 in the NPC group were much higher [29]. Another study showed that the overexpression of MMP3 in NPC epithelial cells increased EBV-induced epithelial cell migration and invasion in an in vitro cell model [30]. One study is aimed at analyzing the co-deregulated genes and their transcriptional regulators in lung cancer [31]. They used a Connectivity Map to find putative repurposing drugs for selected hub genes. Although we also tried to discover the putative repurposing drugs by the same method, the results were not satisfactory. In future research, through more data and deeper research, we plan to complete the unfinished research of discovering the putative repurposing drugs.

## 4. Materials and Methods

### 4.1. Microarray Data

The NCBI-GEO is a public database (https://www.ncbi.nlm.nih.gov/geo/) (accessed on 28 September 2022). By searching for keywords, such as nasopharyngeal carcinogen and Homo sapiens, we obtained three datasets, GSE40290 dataset including 25 NPC tissues and 8 normal tissues, GSE61218 dataset including 10 NPC tissues and 6 normal tissues, and GSE53819 dataset including 18 NPC tissues and 18 normal tissues, which could be downloaded and analyzed by GEO2R.

### 4.2. Identification of DEGs and Data Visualization

We analyzed DEGs between NPC and normal tissues in the GSE40290, GSE61218, and GSE53819 datasets using the GEO2R tool. Volcano and heat maps drawn in each dataset were obtained from the hiplot database (https://hiplot-academic.com/) (accessed on 29 September 2022) [32].

### 4.3. GO and KEGG Pathway Enrichment Analysis of Up- and Downregulated DEGs

DAVID [33] is a web server for gene lists, functional enrichment analysis, and functional annotation (https://david.ncifcrf.gov/) (accessed on 29 September 2022). We used the latest version of the DAVID database (version 7.0) for GO and KEGG pathway enrichment analyses of the upregulated and downregulated DEGs.

### 4.4. PPI Network Construction of Up- and Downregulated DEGs

STRING [34] is an online resource database used to obtain protein association networks (https://string-db.org/) (accessed on 29 September 2022). The content in the database is precomputed and can be downloaded separately by users. We used the 11.5 version of STRING for PPI analysis.

### 4.5. Analyzing the Expression of Hub Genes in Tumor

UALCAN [35] data portal is an interactive network resource (http://ualcan.path.uab.edu/) (accessed on 29 September 2022). The Cancer Genome Atlas (TCGA) transcriptome and clinical patient data were used to study the differential expression of hub genes in normal and tumor tissues through this data portal. And through HPA (https://www.proteinatlas.org/) (accessed on 29 November 2022), protein related data were obtained.

## 5. Conclusions

In conclusion, our analysis identified hub genes and signaling pathways associated with NPC. This provides information for exploring the pathogenesis, identifying molecular targets, and clarifying the biological pathways of NPC. However, further experiments are needed to verify and explore the functions of these genes. Authors should discuss the results and how they can be interpreted from the perspective of previous studies and of the working hypotheses. The findings and their implications should be discussed in the broadest context possible. Future research directions may also be highlighted.

## Data Availability

The data presented in this study are openly available in GEO datasets.
